# Cancer cells invasion to the gastric bare area adipose tissue: a poor prognostic predictor for gastric cancer

**DOI:** 10.1186/s12957-020-02066-5

**Published:** 2020-11-13

**Authors:** Yongming Chen, Shuhang Xu, Chunyu Huang, Yihong Ling, Chengcai Liang, Yuhua Miao, Xiaowei Sun, Yuanfang Li, Zhiwei Zhou

**Affiliations:** 1 State Key Laboratory of Oncology in South China, Collaborative Innovation Center for Cancer Medicine, Guangzhou, China; 2 Department of Gastric Surgery, Sun Yat-sen University Cancer Center, No. 651 Dongfeng Road East, Guangzhou, China; 3 Department of Ultrasound, The Third Affiliated Hospital of Sun Yat-Sen University, Guangzhou, China; 4 Department of Endoscopy, Sun Yat-sen University Cancer Center, Guangzhou, China; 5 Department of Pathology, Sun Yat-sen University Cancer Center, Guangzhou, China

**Keywords:** Gastric cancer, GBA, GBAI, Retroperitoneal infiltration, TDs

## Abstract

**Background:**

The relationship between gastric bare area adipose tissues invasion (GBAI) confirmed pathologically and the prognosis of gastric cancer (GC) patients is undefined. Till present, there has not been literature investigating this phenomenon. Here, we aimed at analyzing the implication of GBAI in GC.

**Methods:**

The data of 1822 patients who underwent radical surgery between January 2000 and December 2013 at the Sun Yat-sen University Cancer Center were retrieved. Pathologically, tumor deposits (TDs) located > 5 mm from the leading edge of the primary tumor and the lymph nodes (LNs) station number 1, 2, 7, and 9 were considered GBAI. Kaplan-Meier method, log-rank test, and Cox’s proportional hazards model were employed to analyze.

**Results:**

Two hundred and five (11.3%) patients were pathologically diagnosed with GBAI, which was more commonly found in proximal or linitis lastica than distal GC (*P* < 0.001). There was significant difference in 5-year survival between patients with and without GBAI for stages IIB, IIIA, IIIB, and IIIC, respectively (*P* < 0.009 for IIB, IIIA, and IIIB; *P* = 0.021 for IIIC). Among the 205 GBAI patients, 61 had detailed radiological follow-up data in which 26 (34.7%) were found to have retroperitoneal infiltration, 27 (36.0%) had peritoneal metastasis, 10 (13.3%) had hematogenous metastasis, 16 (21.3%) had lymphatic metastasis, and 16 (21.3%) had others.

**Conclusions:**

GBAI was identified as a predictor of unfavorable prognosis for GC and was more commonly found in the proximal or linitis plastica of the stomach than in distal stomach. Retroperitoneal infiltration was one of the most commonly identified metastatic route for GC associated with GBAI after radical surgery.

## Introduction

The latest global cancer statistics showed that gastric cancer (GC) is now the sixth most common cancer affecting the global population but still rank third as the leading cause of cancer mortality [1]. In mainland China [2], GC remains third as the most prevalent cancer and leading cause of cancer-related death, illustrating a much higher incidence and mortality than in any other country, and most importantly, China also comprise nearly one half of the total global GC incidence [2–4].

Recurrences and metastases following radical surgery are major challenges to tackle for improving the survival outcomes of GC as these are the main factors associated with GC-related death. Currently, the most effective prognostic factors for GC are the depth of the primary tumor invasion (T), the number of metastatic regional lymph nodes (N), and status of distant metastasis (M), which are merged into known commonly used TNM staging system for GC survival prognostication. However, solely considering the TNM staging system for optimal prognostication may not be reliable as it has been showed that GC patients despite being classified within the same TNM subgroup may still have different survival outcomes, possibly related to tumor heterogeneity [5]. As such, abundant researches have tried to identify other prognostic factors such as vascular invasion, nerve invasion, adipose connective tissue invasion, ratio of lymph node metastasis, and more, but their global acceptance and application has been limited [6–9] as their clinical applicability are controversial and yet to be widely validated. Thus, more researches are still warranted.

The gastric bare area (GBA) is a special location in the stomach without any visceral peritoneum coverage and is located at a small posterosuperior area of the gastric surface, near the cardiac orifice. Specifically, it is the place where the stomach liaise with the diaphragm at the reflections of the gastrophrenic and left gastropancreatic folds, surrounded by the dorsal mesogastrium [10]. From an anatomical point of view, the GBA can be considered a bridge from abdominal organ to retroperitoneal space, saturating with areolar tissue. Till present, there has been only one radiologic study regarding the invasion of the GBA by cancer, termed as the GBA invasion, and was found as a poor prognosis factor for survival [11]. Nevertheless, this study was limited due to considering swollen lymph nodes as one kind of GBA invasion and the absence of pathological evidence. To date, there has been no research regarding the pathological confirmation of gastric bare area adipose tissues invasion (GBAI) and the relationship between GBAI and GC prognosis is yet to be elucidated.

In this present study, we aimed to identify the incidence of GBAI in a large cohort of GC and to evaluate its association with the clinicopathological characteristics and prognosis of GC.

## Materials and methods

### Patients

Between January 2000 and December 2013, the data of 1822 patients who underwent radical surgery at the Department of Gastric Surgery, Sun Yat-sen University Cancer Center, were retrieved. Eligibility criteria for patient inclusion comprised of (1) histologically confirmed diagnosis of gastric adenocarcinoma and R0 gastrectomy, (2) no other synchronous malignancy, (3) no preoperative chemotherapy or perioperative radiotherapy, (4) gastrectomy and lymphadenectomy based on the Japanese Gastric Cancer treatment guidelines [12], (5) more than 15 postoperative pathologically reported lymph nodes, and (6) a postoperative survival time of more than 1 month, considered the non-surgical relative death. Patients with carcinoma of the gastric stump after gastric resection for benign disease were excluded from this study. Retrieved data included the patient gender, tumor size, histological grade, status of vascular invasion, nerve invasion, adipose connective tissue invasion, GBA invasion, depth of invasion (pT), nodal status (pN), distant metastasis (pM), and the number of retrieved lymph nodes. Pathological staging was performed according to the 8th edition of the AJCC cancer staging manual.

### Follow-up protocol

Follow-ups were performed by telephone, email, or outpatient department visits. The last follow-up date was December 2018. The postoperative follow-ups included clinical and laboratory examinations every 3 months for the first 2 years at our outpatient department, every 6 months from the third to fifth postoperative years, and then annually thereafter or until the patient died. Overall survival (OS) was defined as the time from the operation to death or the last follow-up.

### Range of GBAI

**Fig. 1:**
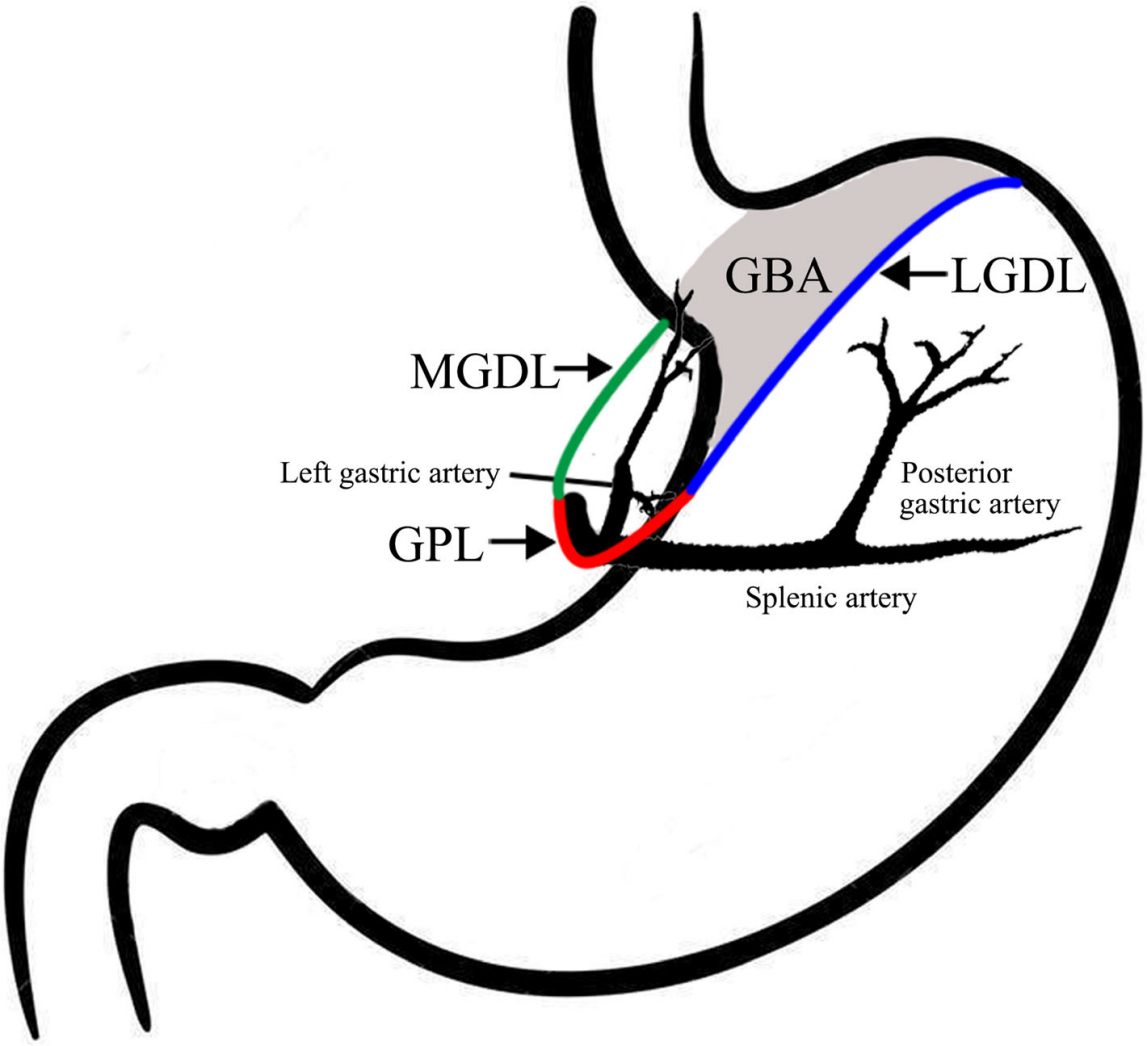
Illustration of GBA, MGDL, LGDL, and GPL

Three important ligaments compose the boundary of the GBA, namely the medial gastrodiaphragmatic ligament (MGDL), lateral gastrodiaphragmatic ligament (LGDL), and gastropancreatic ligament (GPL), as shown in Fig. 1. The hepatogastric ligament contains anterior layer and posterior layer. Rightward extension of the anterior layer becomes the peritoneum of the anterior gastric wall at the lesser curvature. The posterior layer turns posteriorly and right laterally at the lesser curvature, connecting with the right diaphragmatic crura. This peritoneum reflex from the lesser curvature to the right diaphragmatic crura is the so called MGDL which is the right boundary of the GBA. Posterior and right extension of the peritoneum of the posterior gastric wall from the fundus and the great curvature to the left diaphragmatic crura becomes the LGDL, which is the left boundary of the GBA. Inferior extensions of MGDL and LGDL become the GPL at the superior border of pancreas, which is the inferior boundary of GBA. The left gastric artery and vein pass through the GPL. Based on the 3rd English edition of Japanese classification of gastric carcinoma, the definition of the station number 1, 2, 7, and 9 LNs stations are the right paracardial LNs including those along the first branch of the ascending limb of the left gastric artery, the left paracardial LNs include those along the esophagocardiac branch of the left subphrenic artery, LNs along the trunk of the left gastric artery between its root and the origin of its ascending branch, and the LNs along the celiac artery, respectively [13]. Thence, we consider the adipose tissues invasion in the area of LNs station number 1, 2, 7, and 9 or attached to the primary tumor involving the proximal of stomach as GBAI.

### Histologic evaluation of GBAI

For each patient, all postoperative pathological slides were reviewed to evaluate the presence of GBAI by one pathologist who was blinded to the clinical and survival data of patients. Existence of GBAI was examined by reviewing the adipose tissues attached to the primary tumor involving the proximal stomach, and the LNs station number 1, 2, 7, and 9. For pathologic examination of these adipose tissues, tumor deposits (TDs) located > 5 mm from the leading edge of primary tumors and LNs were considered the GBAI, irrespective of their shape, contour, and size.

In this study, adipose tissue invasion was defined as TDs residing in the adipose tissues neighboring the stomach. These TDs were identified using these three primary characteristics: (1) they were located in the adipose tissues and were separated from the primary tumor or lymph nodes; (2) there were no structures of blood or lymphatic vessel, lymphatic nodes, or nerves around them; and (3) they were enveloped by a proper fascia and could be clearly discriminated from peritoneal seeding.

### Imaging assessment

**Fig. 2:**
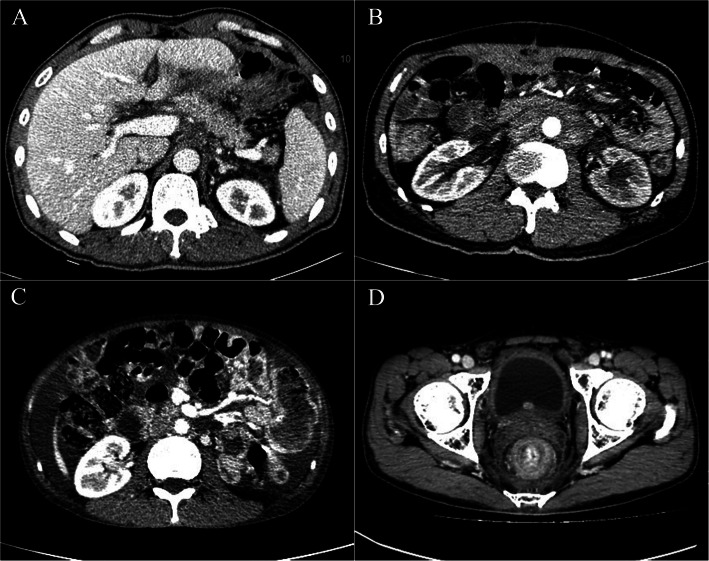
**a** Posterior-pancreas infiltration. **b** Para-aortic infiltration. **c** Mesenterium roots thickening. **d** Thickening of the rectal lining

One radiologist performed all imaging reassessment. She was blinded to the clinical and survival data of patients. CT images were evaluated independently at the workstation using transverse CT (images were reconstructed with a 5-mm section thickness). Metastasis was categorized into five types, including retroperitoneal infiltration, peritoneal, hematogenous, lymphatic, and others. The radiological manifestations containing posterior-pancreas infiltration, para-aortic infiltration, mesenterium roots thickening, and thickening of the rectal lining were considered retroperitoneal infiltration, as shown in Fig. 2; peritoneal nodules/thickening, Douglas pouch nodules, and abdominal mass were considered peritoneal metastasis; liver, lung, and bone were considered hematogenous metastasis; para-aortic, left supraclavicular, mediastinum, and porta hepatis LNs were considered lymphatic metastasis; umbilical region, ovary mass, and anastomotic astium were considered others. It was difficult to classify ascites as retroperitoneal infiltration or peritoneal metastasis, so ascites was considered others.

### Statistical analysis

The 5-year overall survival rate was estimated using the Kaplan-Meier method and univariate comparisons between groups was performed using the log-rank test. In the multivariate analyses, the Cox’s proportional hazards model was carried to estimate the relative risks and to identify corresponding prognostic factors. All data analyses were performed using the SSPS software (version 22.0, Stata Corporation, TX, USA). A *P* value less than 0.05 (two-sided) was considered statistically significant.

## Results

### Clinic-pathological characteristics

Of the 1822 patients, 1220 (67.0%) were male. The mean age of the entire study cohort was 58.0 ± 12.0 years old. The mean number of lymph nodes retrieved was 27.0 ± 10.8. The median follow-up was 92 months (range 3–214 months). The 5-year OS rate for all the patients was 60.9%, and 1109 patients were alive at last follow-up.

**Fig. 3:**
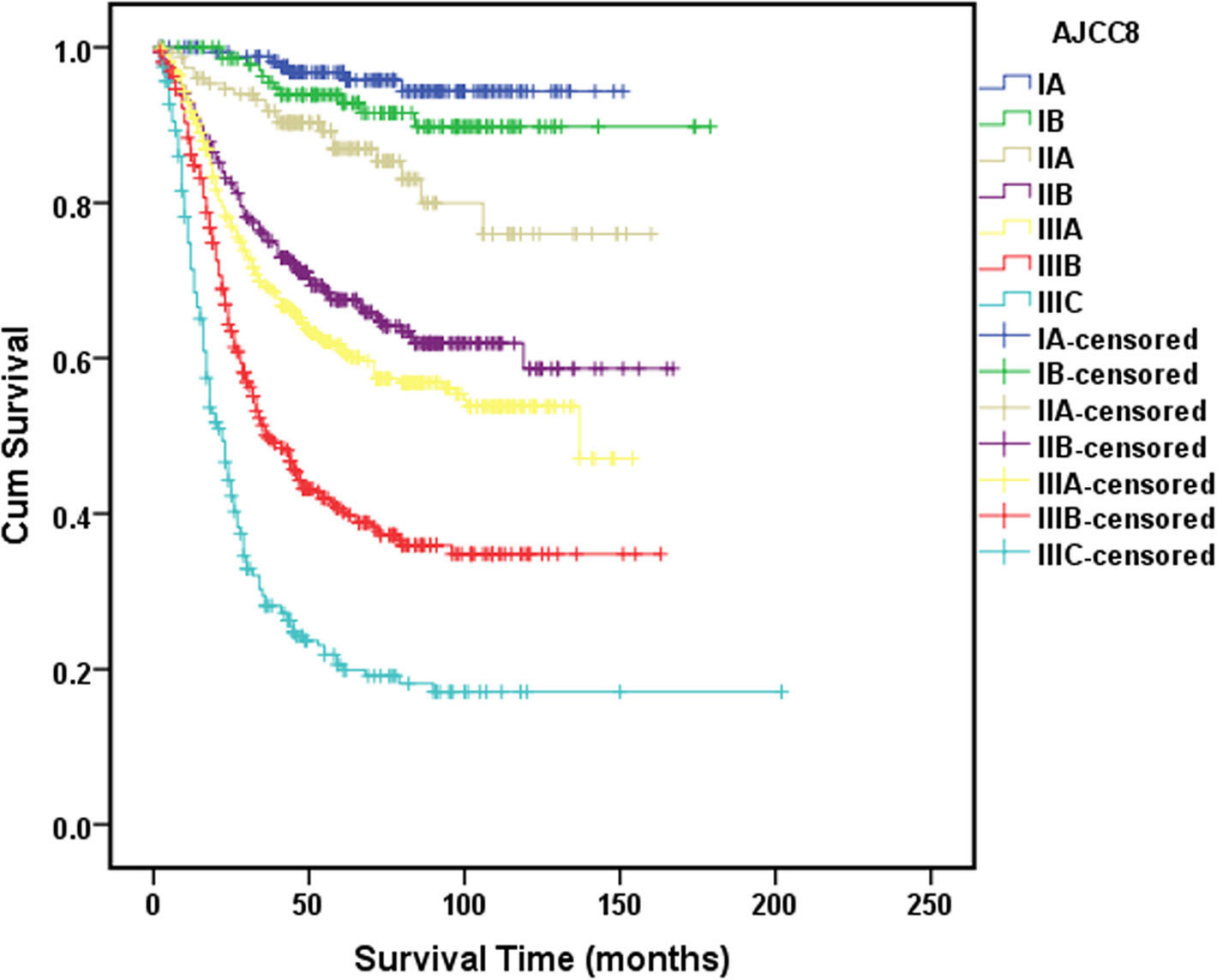
The 5-year survival analysis for all the stages according to the AJCC 8th edition staging system

Eight parameters were significantly associated with the OS on univariate analyses, namely, age, tumor size, tumor site, vascular invasion, adipose tissues invasion, GBAI, depth of invasion (pT), and nodal status (pN) (Table 1). In the multivariate analyses for OS, age, tumor site, adipose tissues invasion, GBAI, depth of invasion (pT), and nodal status (pN) were identified as independent prognostic factors (Table 1). The 5-year survival rates for all the stages were as follows: IA, 96.0%; IB, 92.4%; IIA, 86.2%; IIB, 66.3%; IIIA, 61.1%; IIIB, 43.6%; and IIIC, 25.5% (Fig. 3).

**Table 1:** Clinic-pathological characteristics and the univariate and multivariate survival analysis in gastric cancer patients and in patients with GBAI or not

Variables	*n* (%)	5-year survival rate (%)	*P*	HR (95% CI)	*P*	Non-GBAI (%)	GBAI	*P*	HR (95% CI)	P
Gender										
Female	602 (33.0)	60.0	0.580			534 (33.0)	68 (33.2)	0.883		
Male	1220 (67.0)	61.3				1083 (67.0)	137 (66.8)			
Age (years)										
< 50	478 (26.2)	66.3	0.004	ref.		432 (26.7)	46 (22.4)	0.176		
≥ 50	1244 (73.8)	58.9		1.02 (1.01–1.03)	< 0.001	1185 (73.3)	159 (77.6)			
Tumor size (cm)										
≤ 5.0	1210 (66.4)	66.5	< 0.001	ref.		1112 (68.8)	98 (47.8)	< 0.001	ref.	
> 5.0	612 (33.6)	49.7		1.03 (0.89–1.20)	0.668	505 (31.2)	107 (52.2)		1.08 (0.87–1.33)	0.304
Tumor site										
Distal	1006 (55.2)	70.3	< 0.001	ref.		954 (59.0)	52 (25.4)	< 0.001	ref.	
Proximal/linitis lastica	816 (44.8)	49.3		1.43 (1.23–1.68)	< 0.001	663 (41.0)	153 (74.6)		1.63 (1.40–1.90)	< 0.001
Histological grade										
Well/moderately differentiated	262 (14.4)	69.5	0.234			247 (15.3)	15 (7.3)	0.009	ref.	
Poorly differentiated	1197 (65.7)	58.3				1054 (65.2)	143 (69.8)		1.18 (0.93–1.51)	0.174
Undifferentiated/signet ring cell carcinoma	363 (19.9)	63.1				316 (19.5)	47 (22.9)		1.16 (0.87–1.54)	0.309
Vascular invasion										
No	1529 (83.9)	62.9	< 0.001	ref.		1396 (86.3)	133 (64.9)	< 0.001	ref.	
Yes	293 (16.1)	50.5		0.883 (0.72–1.08)	0.217	221 (13.7)	72 (35.1)		1.07 (0.87–1.33)	0.506
Nerve invasion										
No	1465 (80.4)	60.8	0.932			1348 (83.4)	117 (57.1)	< 0.001	ref.	
Yes	357 (19.6)	61.1				269 (16.6)	88 (42.9)		0.86 (0.69–1.06)	0.152
Adipose tissues invasion										
No	1437 (78.9)	67.6	< 0.001	ref.						
Yes	385 (21.1)	35.6		1.31 (1.05–1.65)	0.019					
GBAI										
No	1617 (88.7)	63.1	< 0.001	ref.						
Yes	205 (11.3)	19.6		1.45 (1.12–1.89)	0.005					
Depth of invasion										
T1	235 (12.9)	96.2	< 0.001	ref.		235 (14.5)	0 (0.0)	< 0.001	ref.	
T2	217 (11.9)	83.4		3.35 (1.61–7.98)	0.001	215 (13.3)	2 (1.0)		3.36 (1.61–7.00)	0.001
T3	421 (23.1)	64.4		5.94 (2.99–11.80)	< 0.001	377 (23.3)	44 (21.5)		6.66 (3.35–13.25)	< 0.001
T4a	892 (49.0)	45.9		8.46 (4.30–16.63)	< 0.001	744 (46.0)	148 (72.2)		9.33 (4.75–18.31)	< 0.001
T4b	57 (3.1)	38.6		10.49 (4.95–22.22)	< 0.001	46 (2.8)	11 (5.4)		10.97 (5.18–23.24)	< 0.001
Nodal status										
N0	561 (30.8)	80.6	< 0.001	ref.		551 (34.0)	10 (4.9)	< 0.001	ref.	
N1	289 (15.9)	74.7		1.08 (0.80–1.46)	0.612	272 (16.8)	17 (8.3)		1.14 (0.84–1.53)	0.400
N2	306 (16.8)	61.1		1.54 (1.18–2.01)	0.001	273 (16.9)	33 (16.1)		1.61 (1.23–2.10)	< 0.001
N3a	402 (22.1)	46.8		2.41 (1.89–3.01)	< 0.001	336 (20.8)	66 (32.2)		2.49 (1.96–3.17)	< 0.001
N3b	264 (14.5)	25.0		3.95 (3.04–5.14)	< 0.001	185 (11.4)	79 (38.5)		4.23 (3.27–5.47)	< 0.001

### Association of GBAI with GC clinicopathological characteristics

Two hundred and five (11.3%) of the 1822 investigated cases were diagnosed with GBAI on pathology, mainly observed in stage IIB, IIIA, IIIB, and IIIC (Table 1). Seven parameters were significantly associated with GBAI on univariate analysis, namely, tumor size, tumor site, histological grade, vascular invasion, nerve invasion, depth of invasion (pT), and nodal status (pN) (Table 1). In multivariate analyses, tumor site, depth of invasion (pT), and nodal status (pN) remained as independent prognostic factors for GBAI (Table 1).

**Fig. 4:**
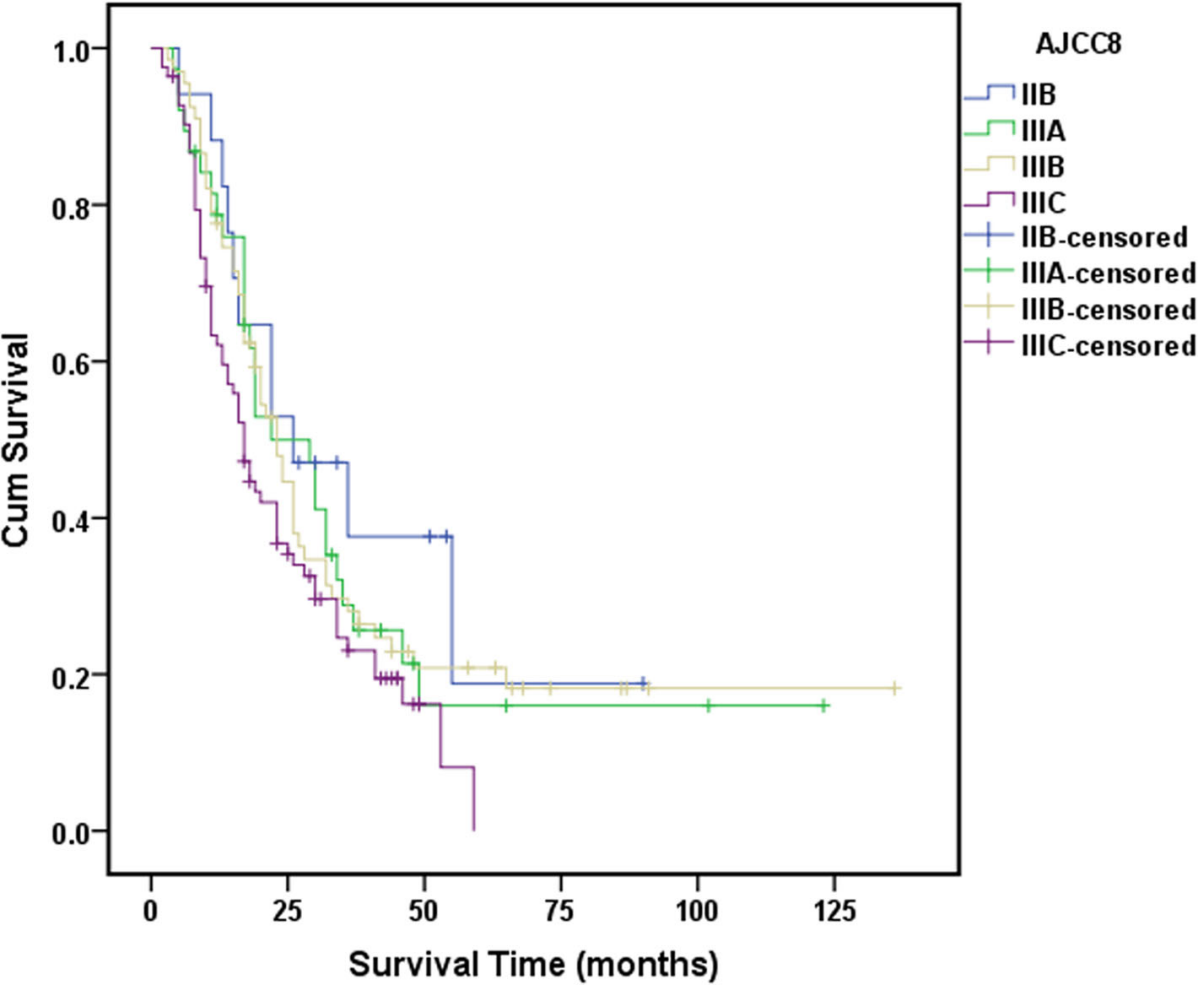
The 5-year survival analysis for GBAI cases

**Fig. 5:**
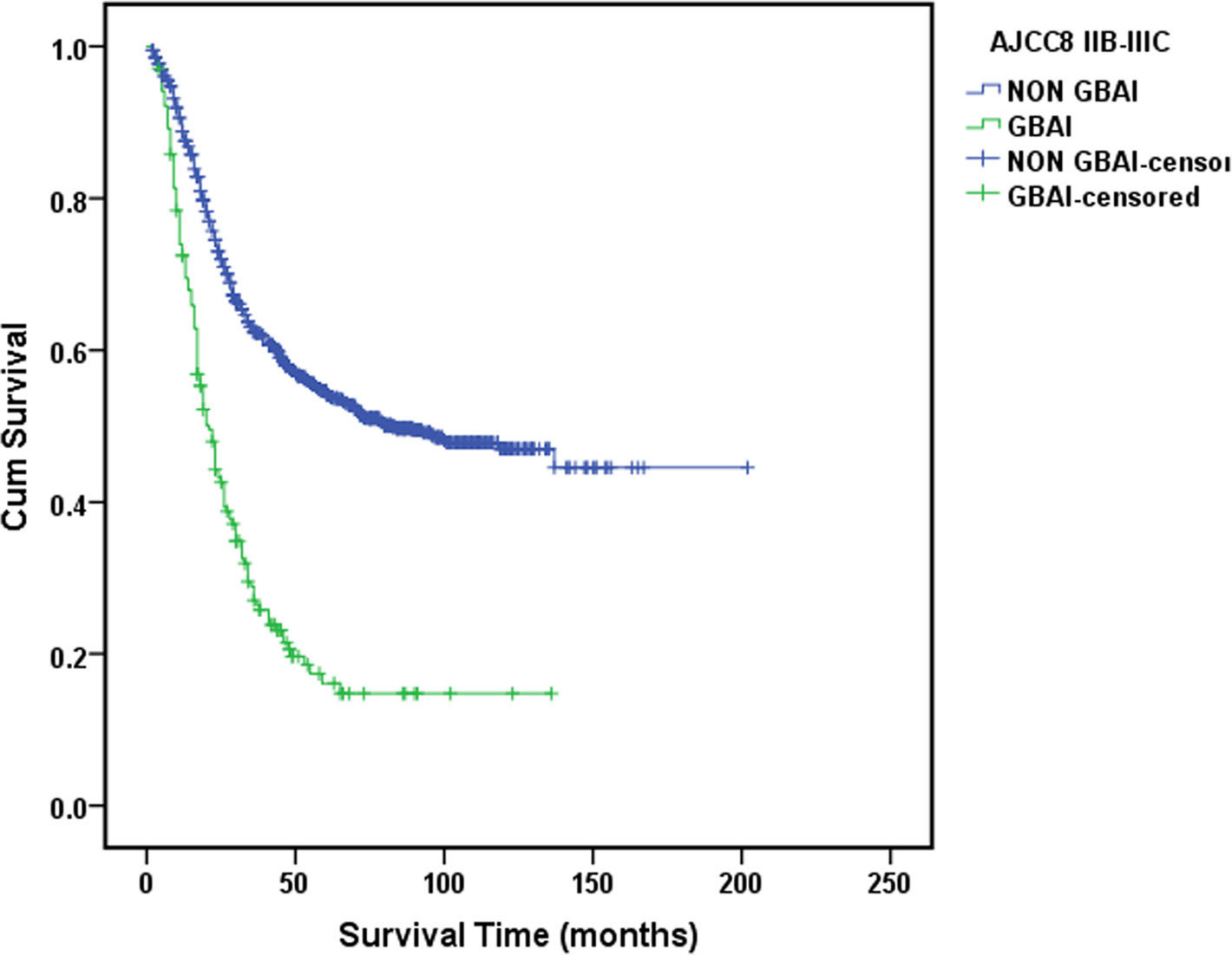
The 5-year survival analysis for GBAI cases and non-GBAI cases in stages IIB, IIIA, IIIB, and IIIC

**Fig. 6:**
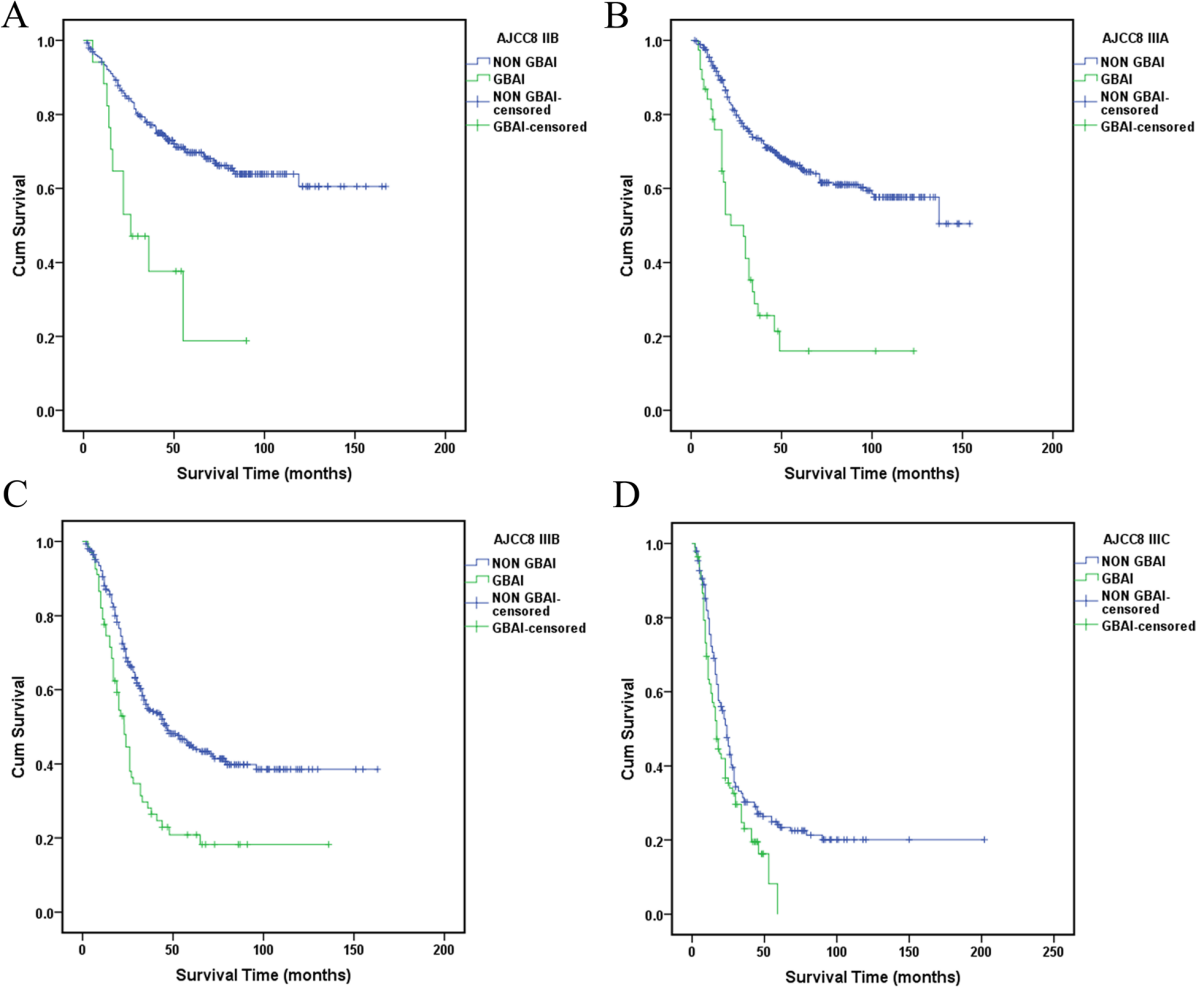
The 5-year survival analysis for patients with GBAI or not in stages IIB, IIIA, IIIB, and IIIC, respectively

**Fig. 7:**
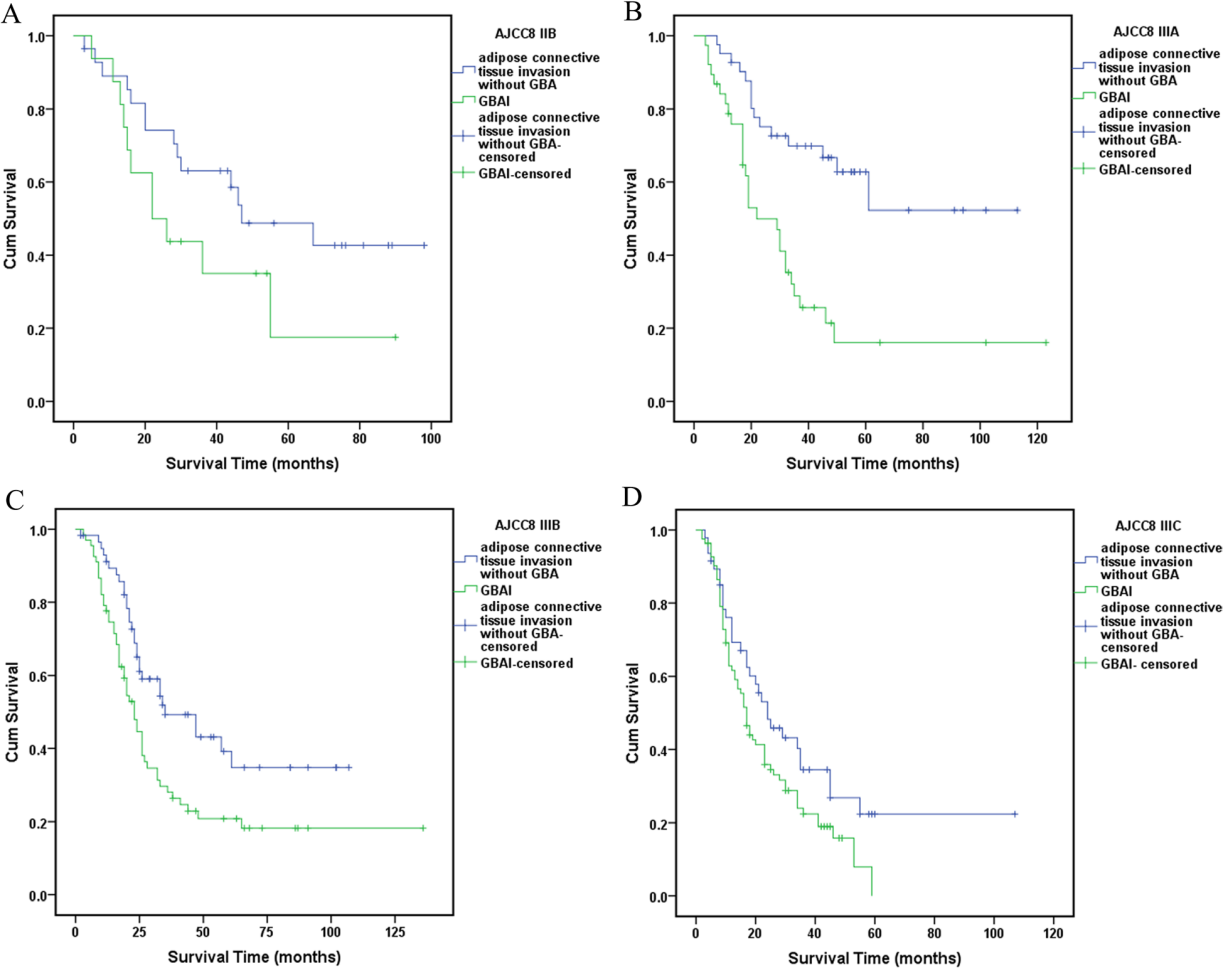
The 5-year survival analysis for patients with GBAI or adipose tissues invasion outside GBA in stages IIB, IIIA, IIIB, and IIIC, respectively

Statistically significant differences in survival were found between all the TNM stage groupings as shown in Fig. 3 (*P* < 0.01). However, no statistically significant difference in survival between GBAI patients in stage IIB to IIIC (*P* = 0.159) (Fig. 4). Interestingly, the difference in 5-year OS between all the patients with GBAI and without GBAI in stages IIB to IIIC was statistically significant (24.4% vs. 54.8%, *P* < 0.01) (Fig. 5). The 5-year OS for patients with GBAI were as follows: IIB, 35.3%; IIIA, 26.3%; IIIB, 23.9%; and IIIC, 21.7%. Further, statistically significant differences between patients with GBAI and without GBAI in stages IIB, IIIA, IIIB, and IIIC were also found (*P* < 0.01 for IIB, IIIA, and IIIB, *P* = 0.021 for IIIC) (Fig. 6). For the purpose of confirming the predictive value of adipose tissues invasion in GBA rather than in other place, we compared the 5-year overall survival rate between these two groups (Fig. 7). The 5-year OS for the patients with adipose tissues invasion outside GBA were as follows: IIB (*n* = 28), 50.0%; IIIA (*n* = 41), 63.4%; IIIB (*n* = 56), 48.3%; and IIIC (*n* = 47), 34.0%; The result showed statistically significant differences in 5-year OS between patients with GBAI and adipose tissues invasion outside GBA in stage IIIA, IIIB, and IIIC, respectively (*P* < 0.01 for IIIA and IIIB, and *P* = 0.050 for IIIC), apart for stage IIB (*P* = 0.117).

### Metastasis status of GBAI patients

In the 205 GBAI patients, only 61 had medical records with detail data of radiological examinations during follow-up (Table 2). Of them, 26 (34.7%) had retroperitoneal infiltration, 27 (36.0%) had peritoneal metastasis, 10 (13.3%) had hematogenous metastasis, 16 (21.3%) had lymphatic metastasis, and 16 (21.3%) had other types of metastasis.

**Table 2:** The detail of metastasis status of GBAI patients

Metastasis paths (*n*)	Radiological manifestations	*n*
Retroperitoneal infiltration (26)	Mesenterium roots thickening	17
	Para-aortic infiltration	4
	Posterior-pancreas infiltration	6
	Rectum circled thickening	7
Peritoneal metastasis (27)	Peritoneal nodules/thickening	18
	Douglas pouch nodules	12
	Abdominal mass	5
Hematogenous metastasis (10)	Liver	7
	Lung	4
	Bone	1
Lymphatic metastasis (16)	Para-aortic	14
	Left supraclavicular	3
	Mediastinum	3
	Porta hepatis	2
Others (16)	Umbilical region	3
	Ovary mass	6
	Anastomotic astium	3
	Ascites	4

## Discussion

Adipose tissues invasion, also known as TDs, is defined as the satellite peritumoral lesion in peritumoral adipose tissue of a primary carcinoma without any histological evidence of any residual lymph node or vessels structure. It was firstly recognized in rectal cancer by Gabriel in 1935 [14]. Quantity of researches confirmed that TDs was not only limited to colorectal cancer but also common to different tumor types, including gastric, biliary duct, and pancreatic cancers [15–18]. The impact of TDs on GC outcomes had been investigated by many researchers and was associated with poor prognosis. In the 8th edition of AJCC gastric cancer staging manual [19], TDs are considered regional LN metastases for the purposes of gastric cancer staging. However, some research suggested that TDs in GC should be incorporated into the T category and treated as a form of serosal invasion [18, 20]. Nevertheless, the prognostic impact of TDs is undeniable, and the algorithm of staging with TDs is controversial.

Our research showed that 205 (11.3%) of 1822 patients were pathologically diagnoses as GBAI and were commonly found in stage IIB, IIIA, IIIB, and IIIC. GBAI status was related to T staging, N staging, and tumor site in multivariate analysis, signifying that deeper gastric wall infiltration and larger number of LNs metastasis could relate to greater risk of identifying GBAI. There are similar survival curves among stage IIB, IIIA, IIIB, and IIIC with GBAI (*P* = 0.159, Fig. 4), and Kaplan-Meier plot showed some overlapping survival curves among each other, implying there is no significant difference in prognosis for patients with GBAI. Upon further analysis, the distance between 5-year survival curves of the patients staged IIB with GBAI and without GBAI, staged IIIA with GBAI and without GBAI, staged IIIB with GBAI and without GBAI, and staged IIIC with GBAI and without GBAI become widely, respectively, reflecting higher mortality risks of the patients with GBAI than without GBAI in the same stage (*P* < 0.009 for IIB, IIIA, and IIIB, and *P* = 0.021 for IIIC, Fig. 6). Significant differences in the 5-year survival rates were also observed between patients with GBAI and with TDs outside GBA in patients staged as IIIA, IIIB, and IIIC (*P* < 0.009 for IIIA and IIIB, and *P* = 0.050 for IIIC, Fig. 7). Although, *P* value of stage IIB is 0.117, with no statistically significant differences, we noted that the distance between two curves spread increasingly wide interval, possibly caused due to the small sample size of stage IIB (*n* = 28 vs. *n* = 16).

The 5-year survival rates for the patients with GBAI were as follows: IIB, 35.3%; IIIA, 26.3%; IIIB, 23.9%; and IIIC, 21.7%. Also, the 5-year survival rates for all patients staged as IIIB and IIIC were 43.6% and 25.5%. The 5-year survival rate of the patients staged as IIB with GBAI was similar to all patients staged as IIIB, and the 5-year survival rates of patients staged as IIIA, IIIB, and IIIC were similar to all patients staged as IIIC. These results demonstrated that GBAI was associated with poorer prognosis than others in the same stage.

From the anatomic point of view, once tumor invade GBA which is a bridge from abdominal to retroperitoneal space, it will spread to the retroperitoneal space subsequently [21]. A mass of researches regarding severe acute pancreatitis [22–30] suggested that there are contiguous spaces in the retroperitoneal spaces which are directly or indirectly related with each other, for instance the peripancreatic region, perirenal and posterior pararenal space, and the root of the small bowel mesentery, as well as pelvic retroperitoneal space. These contiguous spaces are the anatomy basic for retroperitoneal infiltration.

Researches showed that adipocytes, which were found in the close proximity to tumors, along tumor margins, or within the tumor body, exhibited both short- and long-range interactions with cancer cells [31–33]. These adipocytes, the so called cancer-associated adipocytes (CAAs), also referred to as tumor-infiltrating adipocytes, influence tumor biology in a number of ways, including by promoting angiogenesis and inflammation. The CAAs are the molecular biological basic for retroperitoneal infiltration. Hence, cancer cells of patients with GBAI will go through GBA, like a bridge, diffusing to retroperitoneal space under the interactions with CAAs, causing retroperitoneal infiltration which was different from peritoneal metastasis, as shown in Fig. 2. This can be the possible reason of the poor prognosis when cancer invades the GBA. Our follow-up data showed there were 26 (42.6%) cases with retroperitoneal infiltration, which were in accordance with this perspective.

In our research, we found that GBAI in the proximal or linitis plastica GC was more common than distal GC. The GBA is located at the posterior of proximal gastric wall, when the proximal primal tumor penetrates serosa or lymph node metastasis at this area, the cancer cells will invade adipose tissues in GBA easier, being one of the causes for the poor prognosis of proximal GC as compared to of distal GC of the same TNM subgroup. This perspective resonates with Wu et al. [21].

Despite the important findings described, there are still some limitations worth acknowledging in this present study. First, our population cohort was from a single institution, based on relatively limited retrospective data on GBAI, of which only 61 patients had medical records detailing the radiological finding on metastatic route. Second, the basis of the scientific hypothesis of the present study was from clinical observation of retroperitoneal infiltration which is believed to be one of the most commonly identified metastatic route for GC. Nevertheless, future multicenter prospective studies are necessary to validate our findings.

## Conclusions

GBAI was identified as a predictor of unfavorable prognosis for GC and was more commonly found in the proximal or linitis plastica of the stomach than in distal stomach. Retroperitoneal infiltration was one of the most commonly identified metastatic route for GC associated with GBAI after radical surgery.

## Data Availability

The key raw data have been deposited into the Research Data Deposit (http://www.researchdata.org.cn).

## Abbreviations

GC: Gastric cancer
AJCC: American Joint Committee on Cancer
TNM: Tumor node metastasis
TDs: Tumor deposits
GBA: Gastric bare area
GBAI: Gastric bare area tissues adipose invasion
MGDL: Medial gastrodiaphragmatic ligament
LGDL: Lateral gastrodiaphragmatic ligament
GPL: Gastropancreatic ligament
CAAs: Cancer-associated adipocytes
